# Investigation of 9 True Weevil (*Curculionidae* Latreille, 1802) Species for Chitin Extraction

**DOI:** 10.3390/biomimetics9100608

**Published:** 2024-10-08

**Authors:** Zhenying Mei, Luc Vincent, Caroline R. Szczepanski, René-Paul Godeau, Pavel Kuzhir, Guilhem Godeau

**Affiliations:** 1 Université Côte d’Azur, CNRS UMR 7010 INPHYNI, 17 Rue Julien Lauprêtre, 06200 Nice, Francepavel.kuzhir@univ-cotedazur.fr (P.K.); 2 Université Côte d’Azur, CNRS UMR 7272 ICN, Parc Valrose, 06108 Nice, France; 3 Department of Chemical Engineering and Materials Science, Michigan State University, East Lansing, MI 48824, USA; szcz@msu.edu; 4 Université Côte d’Azur, IMREDD, 06200 Nice, France

**Keywords:** *Curculionidae*, pest insects, macromolecules, biopolymer extraction, chitin

## Abstract

Chitin, the second most abundant biopolymer after cellulose, is an important resource for biosourced materials. The global demand for chitin is rapidly increasing, however, the majority of industrial chitin is sourced from crustacean shells, which may be less accessible in regions without seafood waste. Therefore, it is crucial to explore alternative chitin sources, such as those derived from beetles and other arthropods. This study investigated chitin extraction from nine species of *Curculionidae* (true weevils), which are recognized as crop pests. The extraction process and yields were described, and the isolated chitin was characterized by SEM, IR spectroscopy, elemental analysis, XRD, and ash and water content measurements. This work highlights the potential of *Curculionidae* as an alternative chitin source.

## 1. Introduction

In a world where climate change is expected to have a considerable impact on the ability of humans to produce food, invasive insects and pests that can significantly harm or destroy vegetable production are of particular concern. Invasive insects are often responsible for the complete destruction of an agricultural culture, and thus it is very important to prevent these events with different treatments. Prevention may include different control strategies including: physical, chemical, biological, or cultural methods [1,2]. But if prevention fails, an important part of the production is lost. Considering these challenges, it is of interest to identify resources that can be produced or extracted from invasive species, and the mechanisms by which this extraction may be feasible. Ultimately, identifying strategies to create value from invasive species that cannot be eliminated easily would have a significant impact.

One interesting method to obtain a valuable product from invasive insect species would be through chitin extraction. Insects are arthropods, and as a consequence, they have an exoskeleton rich in chitin. Chitin is a chain polymer of *N*-acetylglucosamine and is the second most abundant polysaccharide polymer after cellulose [3]. In Arthropoda’s exoskeleton and as consequence in insect’s exoskeleton, the most abundant form of chitin is the α form. In this form, chitin presents a fully anti-parallel organization. This organization leads to many hydrogen bonds and, as consequence, high stability and low solubility [4].

**Figure 1:**
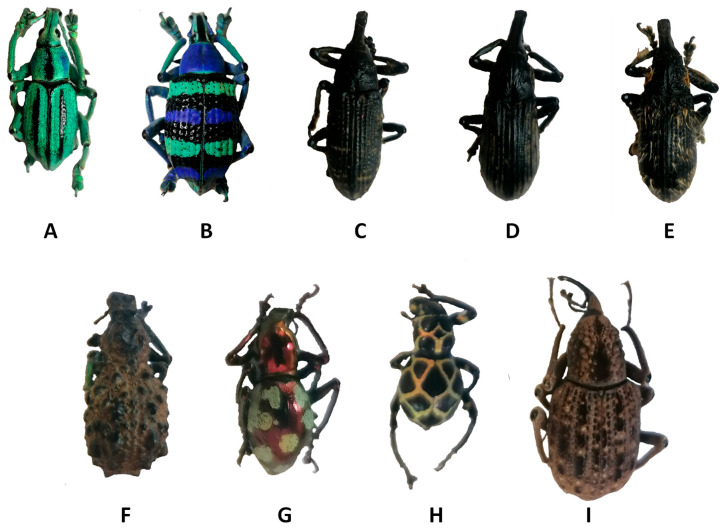
Examples of *Curculionidae* specimens. (**A**) *Eupholus cuvieri* Guérin-Méneville, 1830, (**B**) *Eupholus magnificus* Kirsch, 1877, (**C**) *Lixus sturmii* Boheman, 1836, (**D**) *Lixus gigas* Fairmaire, 1904, (**E**) *Lixus albicornis* Fairmaire, 1904, (**F**) *Holonychus saxosus* Coquerel, 1859, (**G**) *Pachyrhynchus gemmatus purpureus* Kraatz, 1888, (**H**) *Pachyrhynchus reticulatus* Waterhouse, 1841 and (**I**) *Sipalinus gigas* Fabricius, 1775.

Unfortunately, due to this poor solubility in most common solvents, α-chitin is difficult to use in materials applications in its pristine form. This lack of solubility is a consequence of strong intermolecular interactions and a compact macromolecular structure. Typically, to avoid this solubility limitation, chitin is modified prior to industrial use. For example, chitin is the main raw component in chitosan production once it is partially deacetylated. Chitosan is an important material that can be used for many application domains including food, medicine, and textiles [5,6,7,8,9,10,11,12]. Chitin-sourced chitosan is already produced on an industrial scale, mainly from seafood waste as the exoskeletons of shrimp, lobster and crabs are rich in chitin [13,14,15]. Due to the human consumption of crustaceans, this is a relatively large resource. Despite this, and due to the important industrial interest, new chitin sources are investigated. Recent works highlight that mushroom, corals, sponges and terrestrial Arthropoda are promising sources of chitin [16,17,18,19,20,21,22,23]. Our prior work investigated beetles (giant flower beetles or dung beetles) as a source of chitin and therefore chitosan [24,25,26]. Building upon this foundation, here we focus on the possibility to use *Curculionidae* as a potential source of chitin (Figure 1). This includes extensive SEM observation of the specimens prior to and following chitin extraction, and the extracted chitins are fully characterized. *Curculionidae* include a great diversity of species including some species that are considered pest insects. For example, true weevils cause havoc in cocoa, palm, sugar beet and banana productions [27,28,29,30,31,32]. Of course, not all *Curculionidae* are classified as pest insects, and some are beneficial as pollinators. For this study we chose various species from different genus (including pest and beneficial species) to determine the best candidates for chitin extraction, which can inform future strategies for materials development.

## 2. Materials and Methods

### 2.1. Materials

The specimens used in this work came from the private collection of G. Godeau. All observations were performed on dried samples of dead specimens. No specimens were sacrificed for this work. Due to the small size of some species studied, such as animals of the genus *Pachyrhynchus*, it is difficult to separate the internal elements and exoskeleton of the specimens. Therefore, all specimens were used whole to have the same treatment for all species. The internal elements are hydrolyzed, degraded and eliminated during chemical treatment. To consider the diversity of the *Curculionidae* family, 9 species belonging to 5 genera (including *Lixus* and *Sipalinus*) were investigated in this work. With this design, both beneficial and pest insects are considered. The complete list of the studied species is reported in Table 1.

### 2.2. Chitin Extraction [24]

Demineralization: Dry samples were immersed in 1 M HCl aqueous solution. The solution was then heated for 2 h (95 °C) using a dry bath. The liquid phase was filtered off, and the resulting exoskeleton was rinsed with deionized water until reaching a neutral pH. The exoskeleton was then dried in an oven overnight at 90 °C to estimate the demineralization yield:

(1) $$\mathrm{Demineralization}\;\mathrm{yield}=\frac{\mathrm{Demineralized}\;\mathrm{mass}}{\mathrm{raw}\;\mathrm{mass}}\;*\;100$$

The exoskeleton was used for deproteination without any further purification.

Deproteination: After demineralization, the exoskeletons were placed in an aqueous 2 M NaOH solution. The solution was then warmed (95 °C) over a period of 36 h using a dry bath. During this treatment, the solution rapidly turned black. Therefore, the NaOH solution was refreshed hourly during the first 6 h of the treatment. The liquid phase was then removed, and the exoskeletons were rinsed with deionized water until achieving a neutral pH. The exoskeletons were then dried in an oven overnight at 90 °C to determine the deproteination yield:

(2) $$Deproteination\;yield=\frac{Deproteinated\;mass}{Demineralized\;mass}\;*\;100$$

The resulting material is directly used for bleaching without further purification.

Bleaching: The deproteinated exoskeletons were bleached using an aqueous solution of sodium hypochlorite (3.6 wt. %) at room temperature for 1 h. The bleached exoskeletons were then washed multiple time with deionized water and then with ethanol. The samples were finally dried in an oven (90 °C). The overall yields are determined as:

(3) Overall yield=Demineralization yield ∗ Deproteination yield

All yields are presented in Table 2.

### 2.3. Chitin Characterization

#### 2.3.1. FT-IR Characterization

Fourier Transform Infrared spectroscopy (FT-IR) measurements were carried out using a Spectrum Two FT-IR spectrometer from Perkin Elmer with universal ATR accessory. The measurements were performed between 4000 cm^−1^ and 500 cm^−1^.

#### 2.3.2. Thermal Analysis (TGA)

Thermogravimetric (TGA) measurements were performed on a TGA/DSC 1 from Mettler Toledo. The samples were heated from 25 °C to 850 °C with a heating rate of 10 K.min^−1^ under nitrogen flow of 50 mL.min^−1^, the gas was then switched to air to oxidize carbon and determine the ash content.

#### 2.3.3. X-ray Diffraction (XRD) Analysis

X-ray diffraction of powdered chitin samples were examined by a Panalytical X’Pert Pro with an Xcelerator fast detector operating at 45 kV and 30 mA. The radiation was generated from a Cu Kα (k = 0.15418 nm) source. The diffraction data were collected at 2*θ* values from 5° to 75°.

The crystallinity indices of isolated chitosan samples (CrI) were calculated from XRD data using the following equation [19,33]:

(4) $$CrI=\frac{I_{cr}-I_{am}}{I_{cr}}\;*\;100$$

where *I_cr_* is the maximum intensity for crystalline lattices at 2*θ* = 19.6° and *I_am_* is the maximum intensity at 2*θ* = 16°, corresponding to the amorphous region.

#### 2.3.4. Elemental Analysis

Elemental analyses were carried out on an elemental analyzer Flash EA 1112 series (Thermo Finnigan, Waltham, MA, USA), equipped with Eager 300 Xperience software.

#### 2.3.5. Scanning Electron Microscopy

SEM observations were carried out using Phenom ProX scanning electron microscope. Samples were observed with a gold coating at an accelerating voltage of 5 and 10 kV. The samples were coated using Q150R S Sp.

## 3. Results

**Figure 2:**
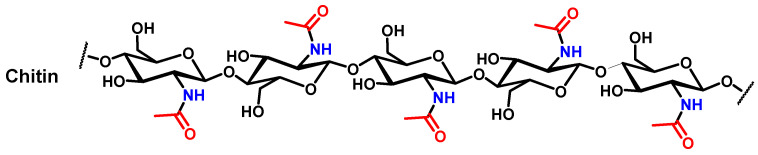
Theoretical chemical structure of chitin.

*Curculionoidae*, also known as true weevils, are one of the most diverse groups of phytophagous *Coleoptera*. More than 51,000 species belonging to approximately 4600 genera of *Curculionidae* have been described [34]. Among this wide diversity, many weevils can be described as pest insects that harm various crops or ornamental plants including banana, cocoa, and palm [27,28,29]. For example, the *Lixus* genus is reported as major pests of sugar beet in Iran and leafy vegetables in Nigeria. Another example, *Sipalinus gigas*, are considered as one of the most significant wood pests in Japan [31,35,36]. All the specimens were treated for chitin extraction. The selected chitin extraction strategy is the classical chemical approach described for many decades for chitin extraction from shrimp [24,37]. It consists of a three step treatment that can be summarized as follows. The first step entails demineralization in an HCl solution (1 M in water) for 1 h at 95 °C. The second step consists of immersion in a sodium hydroxide solution (2 M in water) at 95° over 36 h. The third and final step is bleaching. For this bleaching step, the material is immersed in a sodium hypochlorite solution (3.6 wt. % in water) for 1 h at room temperature. Between each step, the material is washed with water. The final isolated material can be reported as *Curculionidae*’s chitin. The extracted material should theoretically present a chemical structure similar to the one presented in Figure 2.

Of course, the selected species went through the chitin extraction process separately to obtain species-specific data. All the data collected at each stage of the extraction are reported in Table 2, including yields for demineralization, deproteination and the overall yield of both steps.

The chitin extraction data mostly show uniform results. The demineralization step has an associated yield between 80 and 90%. For deproteination, the yield is roughly 15–25%, and the overall yield is between 12 and 19%. These values remain similar for all species. Compared with chitin extraction yields reported for other *Coleoptera* in the literature, the values are consistent but remain in the low range of reported values [22]. These low yields may be a consequence of the use of whole specimens that lead to underestimation of the overall yield. However, even if the extraction yields are in a low range compared with other beetles, these yields remain significant compared to the yield for chitin extraction from shrimps.

The extracted samples of chitin were then characterized. All treated surfaces were first investigated for their morphologies, using scanning electron microscopy (SEM) and compared with the corresponding virgin surfaces. Not surprisingly, depending on the species, the surface morphologies were very different. For example, some *Curculionidae* genera are known to exhibit structural coloration like *Eupholus* (Figure S1) and *Pachyrhynchus* (Figure S2).

For both genera, the surface morphology varies across the specimens. For example, the colored parts (blue or green) of *E. cuvieri* (Figure S1A) and *E. magnificus* (Figure S1C) show nanostructured (wrinkled) microscales which contribute to the structural color, as has been previously described in the literature [38]. Not surprisingly, the black part of both species, which lack any structural color, is smooth when observed via microscopy (Figure S1B for *E. cuvieri* and Figure S1D for *E. magnificus*). Similar microscales are observed for *P. reticulatus* and *P. gemmatus purpureus*. Here, these microscale structures correspond to the golden network present on *P. reticulatus* (Figure S2A) and the large green spots on *P. gemmatus purpureus* (Figure S2C).

On these species, other types of surface are observed, both the black part of *P. reticulatus* (Figure S2B) and the metallic red surface of *P. gemmatus purpureus* (Figure S2D) are smooth compared with the previous one. Of course, the red metallic surface of *P. gemmatus purpureus* is structurally colored but the structuration remains under the surface. For the *Lixus* genus, all species present similar surface morphologies. These surfaces are highlighted in the supporting information in Figure S3. For *Lixus* species, most of the observed surfaces are smooth, with or without hairs depending on the position imaged (Figure S3). Observations of *H. saxosus*, which lacks any structural coloration, reveals dense microscale organizations (Figure S4A,B). The surface of ***S. gigas*** has vertically aligned pins (Figure S4C,D). This structuration appears to be quite uniform along the darkest surface.

**Figure 3:**
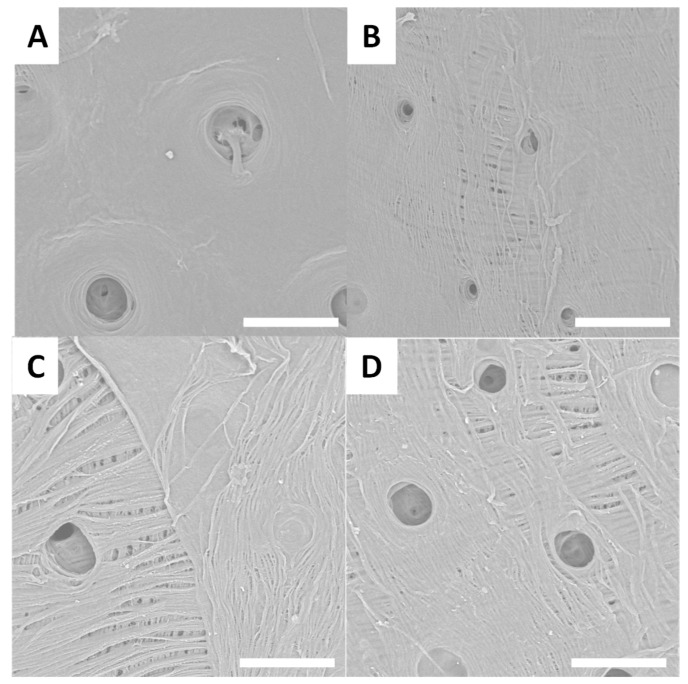
Examples of SEM images (scale bar = 30 µm) observed for treated surfaces of *E. cuvieri* (**A**,**B**), *E. magnificus* (**C**,**D**).

**Figure 4:**
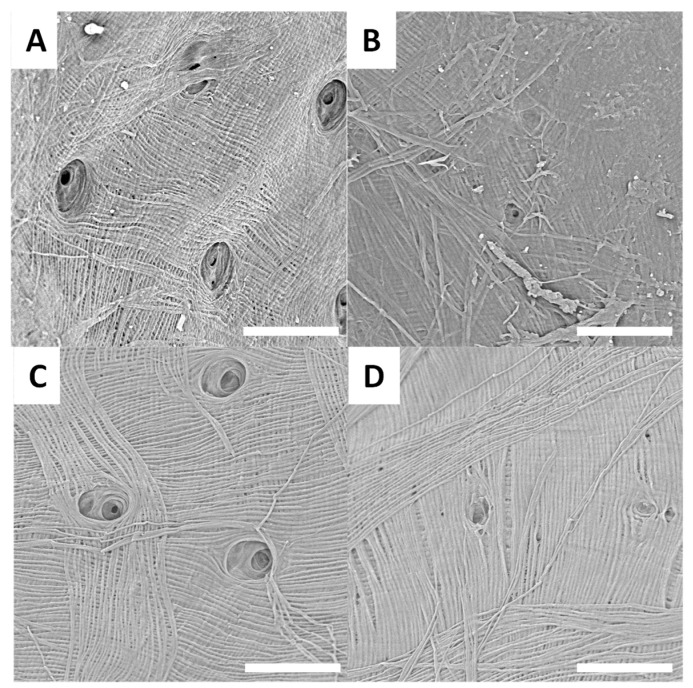
Examples of SEM images (scale bar = 30 µm) observed for treated surfaces of *P. gemmatus purpureus* (**A**,**B**) and *P. reticulatus* (**C**,**D**).

**Figure 5:**
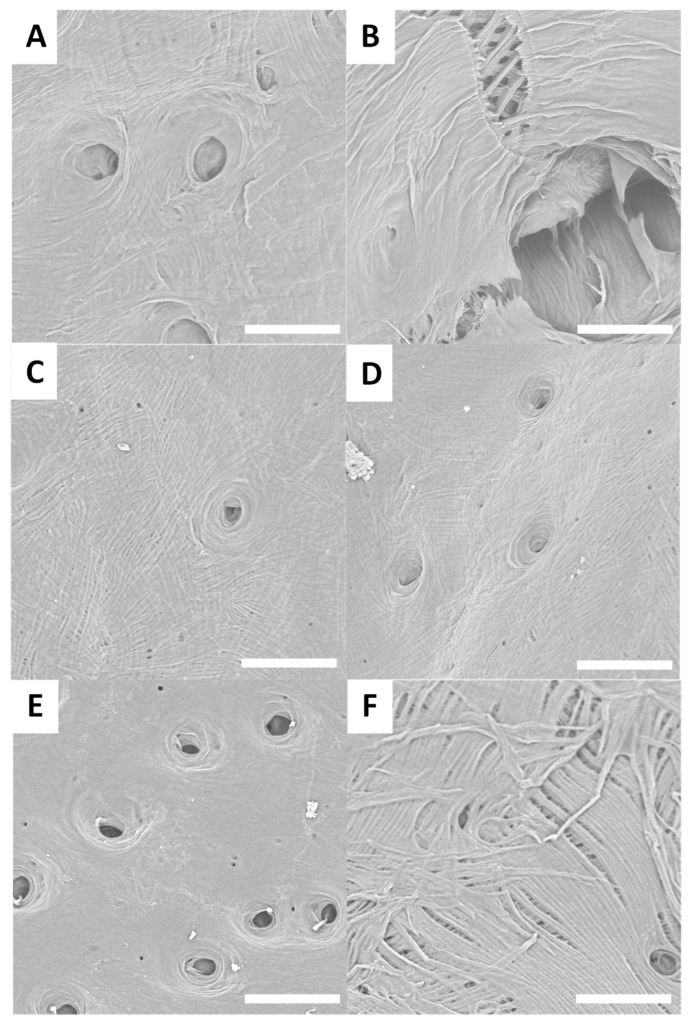
Examples of SEM images (scale bar = 30 µm) observed for treated surfaces of *L. sturmii* (**A**,**B**), *L. gigas* (**C**,**D**) and *L. albicornis* (**E**,**F**).

**Figure 6:**
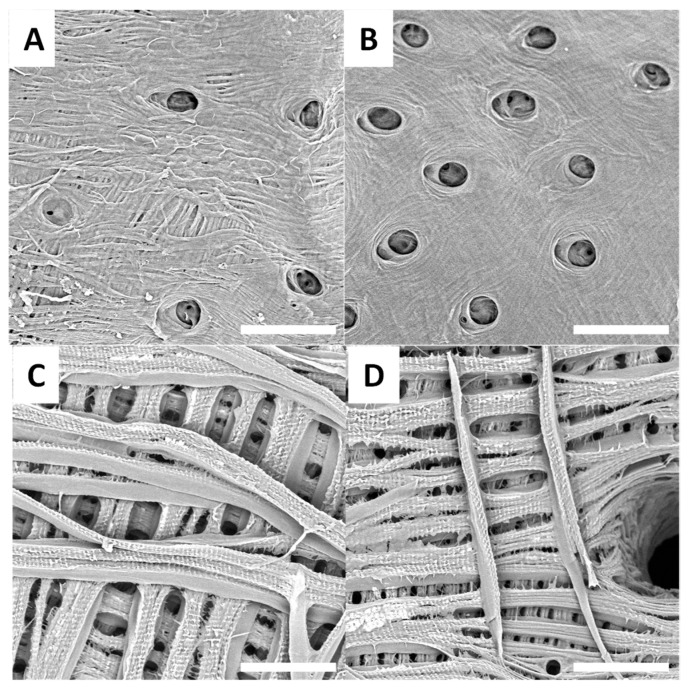
Examples of SEM images (scale bar = 30 µm) observed for raw surfaces of *H. saxosus* (**A**,**B**) and *S. gigas* (**C**,**D**).

After the treatment used to extract chitin, surfaces reveal an inner-connected chitin network (Figure 3, Figure 4, Figure 5 and Figure 6). SEM images presented in Figure 3A–D show treated surfaces from *Eupholus* species. The surface morphology can provide valuable insights into the effectiveness of the extraction process. Differences in surface structure between species can affect the efficiency of deproteinization and demineralization, and SEM helps to visualize these effects. Additionally, the observation of different surface morphologies corresponding to various insect surface colors provides insights that could contribute to bionics research. These differences in morphology may have implications for bioinspired design, offering potential applications beyond chitin extraction.

SEM images of *Pachyrhynchus* species’ treated surfaces are presented in Figure 4A–D.

Different *Lixus* species surfaces after treatment are presented in Figure 5A–F.

Treated surfaces from *H. saxosus* are presented in Figure 6A,B, and treated surfaces from *S. gigas* are presented in Figure 6C,D.

SEM observations of the treated surfaces reveal holes on most of them. These holes can be explained by the loss or degradation of insects’ surfaces microstructures during the chemical treatment. In the case of treated surfaces from *Eupholus*, *Pachyrhynchus* and *Holonychus* species, the holes may be linked to the loss of the scales from raw surfaces. These observations are consistent with the progressive loss of structural coloration seen during treatment, especially for *Eupholus* species. In the case of treated surfaces from *Lixus* species, the holes maybe linked to the degradation (during treatment) of the air shown by the raw surfaces. As the *Sipalinus* does not present such kind of microstructures, no holes were observed on the corresponding treated surfaces.

For most of the treated surfaces, a fiber network is observed even if the network has qualitative differences from one specie to the other. If such kind of network are consistent with expected structures for chitin materials, it remains only superficial observations. To investigate these variations in surface characterization further, additional experiments are needed.

FT-IR analyses were performed on all extracted chitin samples. Examples of FT-IR spectra for the select species are shown in Figure 7, and all other FT-IR spectra collected are provided in the Supplementary Data (Figures S5–S12).

The FT-IR spectra for each extract have results consistent with chitin material. For example, strong bands at 3380–3450 cm^−1^ and 3250–3300 cm^−1^ are observed, consistent with the stretching of O-H (red) and N-H bonds (green), respectively. Additionally, a band corresponding to sp^3^ CH_2_ vibration is observed at 2850–2950 cm^−1^ (blue), and the C=O band from amide group is observed at 1635–1660 cm^−1^ (orange). The bending and vibration bands from N-H and stretching band from C-N are observed at 1560–1580 cm^−1^ (purple). Finally, a deformation band of CH_2_ is reported at 1410–1425 cm^−1^ (grey). This first observation is consistent with bands expected for chitin. However, to conclude on the chitin form isolated from *Curculionidae*, IR observation should be more detailed. All observed IR bands are presented in Table 3.

Comparison of IR observed for extracted chitin, *α* -chitin (anti-parallel organization) and *β*-chitin (parallel organization) allows the identification of the chitin form from Curculionidae’s species. Some bands are of particular interest. In particular, the split C=O bands observed between 1635 and 1660 cm^−1^ are characteristic of the *α*-form compared to the *β*-form that shows only one band. Additionally, the displacement observed for the O-H, N-H, CH_2_, N-H and C-N bands are closer from the *α*-chitin instead of the *β*-chitin. For bands from 850 cm^−1^ to 1400 cm^−1^ differences are more difficult to distinguish. However, these differences are enough to suggest that the extracted chitin form is the *α*-chitin [39]. This observation is consistent with the literature that describes the chitin from most coleoptera as *α*-chitin.

The extracted materials were also characterized using thermal gravimetric analysis (TGA). Examples of TGA curves are presented in Figure 8 (data for other species are presented in the Supplementary Materials, Figures S13–S21).

TGA is used here to determine thermal degradation temperature, then the ash and water content. The ash content is related to the remaining mineral fraction of the extracted chitin and the water content corresponds to the amount of water spontaneously trapped in the material (Table 4).

The TGA data reveal a degradation temperature greater than 350 °C for all extracted chitins. This is consistent with temperatures reported in the literature for *α*-chitin [40]. For most species, the ash content is reported near or below 5% except for *P. gemmatus purpureus*, which is higher (9.7%). All these values remain consistent with values previously reported for beetles in the literature [22]. Regarding the measured water content (moisture content), the reported data are from 2 to 5%.

The extracted chitin was also characterized using elemental analysis. All elemental analysis data are reported in Table 5 and are consistent with theoretical values considered for chitin.

**Figure 9:**
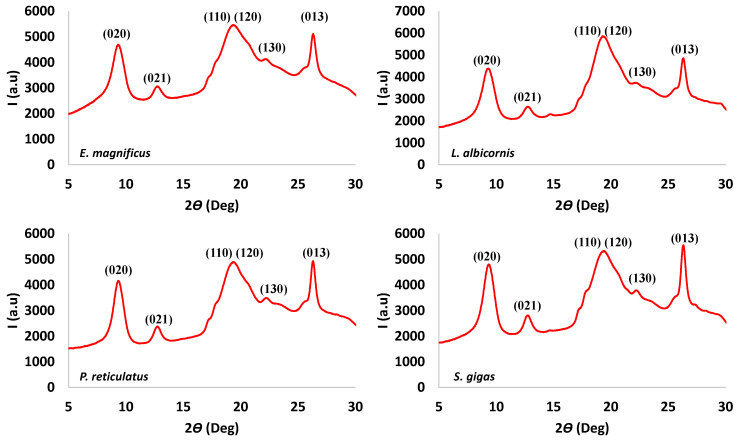
Examples of X-ray spectra observed for *Curculionidae* specimens.

Finally, the extracted chitins were evaluated using X-ray diffraction. Examples of the X-ray observations are shown in Figure 9, and data for the remaining species are provided in the Supplementary Data.

All chitin samples have similar XRD patterns, which are at 2*θ* values of 9.4°, 12.9°, 19.6° shouldering with 20.6, 22.3° and 26.4°. These data were compared with XRD patterns reported for *α* and *β* chitin (Table 6).

Most of the values reported for extracted chitins are consistent with the *α*-form. Those results confirm the observation made with the IR and thermogravimetric data. Considering the extracted chitin as α-chitin, it is possible to hypothesize that the observed rays may be respectively attributed to the plane (020), (021), (110) shouldering with (120), (130) and (013) [39]. The crystallinity results for the extracted materials are presented in Table 7.

For all species, the crystallinity index is between 45 and 60%. This index can be described as low compared with crystallinity index reported in the literature for other coleopters [19,22]. With all the performed characterizations, it is concluded that the extracted material has similar characteristics to classical shrimp chitin which is also *α*-chitin. The average overall yield near 20% for *Curculionidae* is comparable (even if in the low range) to other beetle chitin yields [22]. However, compared with shrimp, the yield is greater. As consequence, it is reasonable to consider *Curculionidae* as a potential source of chitin for future industrial exploitation.

## 4. Conclusions

As a conclusion of this work, we report here for the first-time chitin extraction from nine *Curculionidae* species from five different genera. Chitin was obtained using simple, straight-forward chemical treatments including demineralization, deproteination and bleaching. After the three-step process, the treated cuticles produced chitin with yield comprised between 12 and 20%. The observed yield can be reported as low compared to other beetles, but remains high if compared to chitin extraction yields for shrimp. The extracted chitins were characterized for all species using various techniques including SEM, FTIR, TGA, X-Ray and elemental analysis.

SEM observations show important modification of the materials after treatment. All surfaces present a fibrous network with different shapes. As the extracted chitin needs to be processed to reach chitosan, the morphology differences should not have an impact for further material application. FTIR and X-Ray observations reveal indeed that the extracted material can be described as chitin but also that the extracted chitin is α-chitin. As for FTIR and X-Ray, the elemental analysis gives values very similar for all extracted chitins. Those values are also consistent with expected values for chitin. Thermogravimetric analysis revealed that extracted materials present ash and water contents around 3 to 10% and 2 to 5%, respectively, but also that the extracted materials present high degradation temperature (greater than 350°). In summary, all the characterizations are consistent with chitin and, more precisely, with *α*-chitin. Except for the morphological observations, the presented data show no significant difference, depending on the studied species. As a consequence, it is possible to assume that all studied *Curculionidae* species are suitable for chitin extraction. These preliminary data are of particular interest and demonstrate that ravaging animals may have an application as a potential source of chitin for industrial uses and that pest insect invasion may be considered as an underestimated resource of biosourced material elaboration.

## Figures and Tables

**Figure 7:**
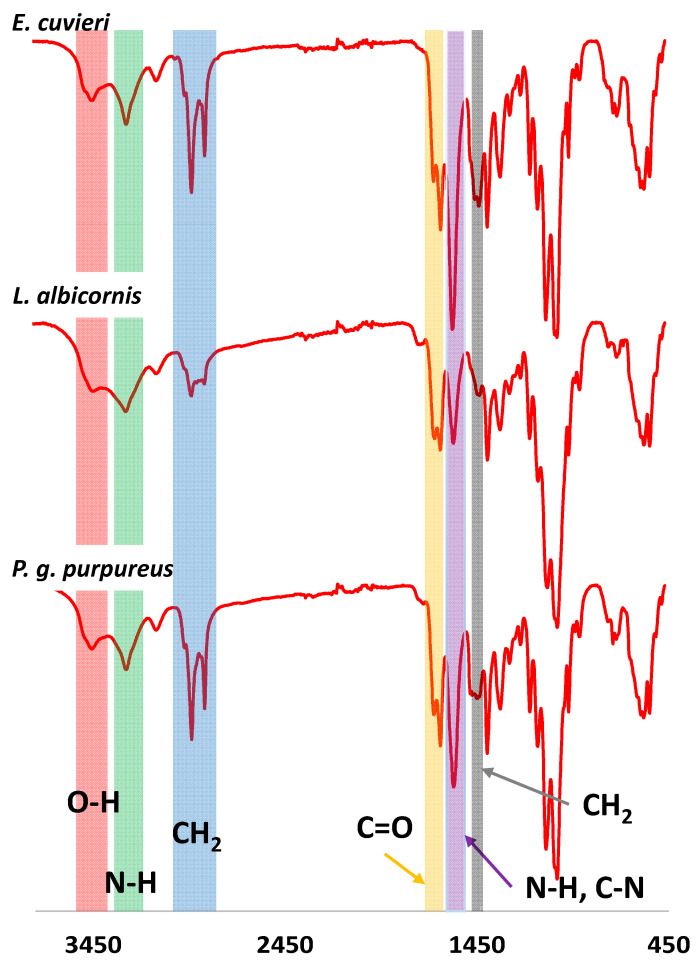
Examples of FTIR spectra observed for *Curculionidae* specimens.

**Figure 8:**
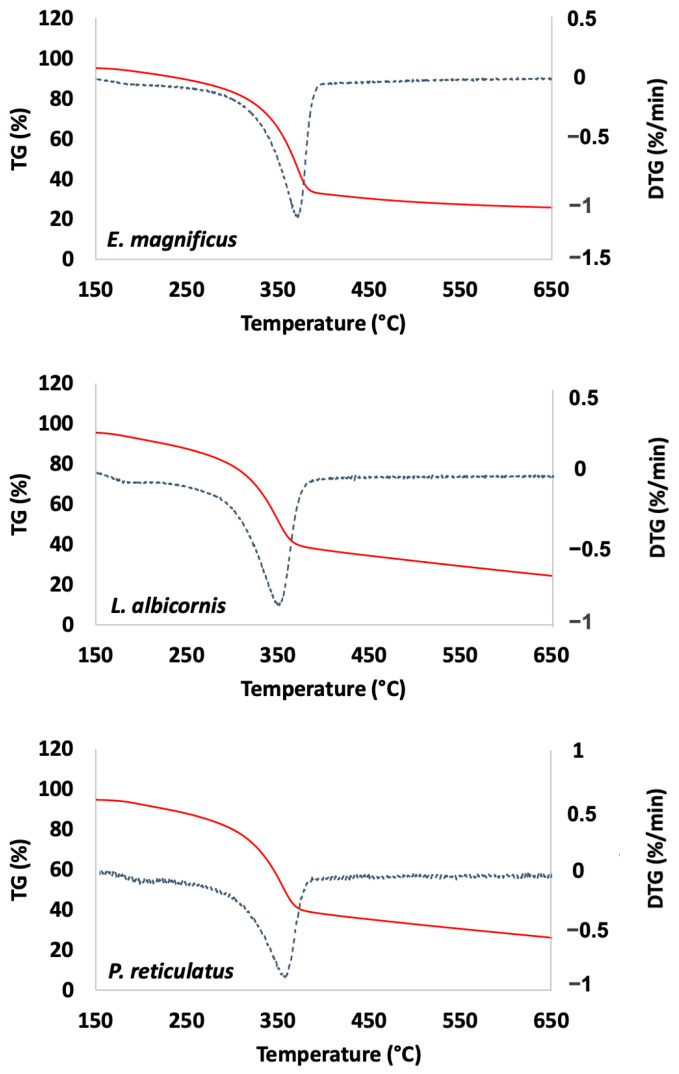
Examples of thermal analysis observed for *Curculionidae* specimens (TG: red and DTG: blue).

**Table 1:** Studied species data.

Studied Species	Descriptor	Specimens Collection Area
*Eupholus cuvieri*	Guérin-Méneville, 1830	Indonesia
*Eupholus magnificus*	Kirsch, 1877	Indonesia
*Lixus albicornis*	Fairmaire, 1904	Madagascar
*Lixus gigas*	Fairmaire, 1904	Madagascar
*Lixus sturmii*	Boheman, 1835	Madagascar
*Holonychus saxosus*	Coquillet, 1859	Madagascar
*Pachyrhynchus reticulatus*	Waterhouse, 1841	Philippines
*Pachyrhynchus gemmatus*	Kraatz, 1888	Philippines
*Sipalinus gigas*	Fabricius, 1775	Australia

**Table 2:** Chitin extraction data.

Species	Demineralization Yield (%)	Deproteination Yield (%)	Overall Yield (%)
*Eupholus cuvieri*	84.7	19.9	16.7
*Eupholus magnificus*	85.8	22.6	19.3
*Lixus albicornis*	78.1	21.2	16.4
*Lixus gigas*	84.6	19.2	15.9
*Lixus sturmii*	84.9	21.2	18.0
*Holonychus saxosus*	84.9	18.4	15.5
*Pachyrhynchus reticulatus*	86.5	15.5	13.5
*Pachyrhynchus gemmatus*	80.6	15.2	12.1
*Sipalinus gigas*	94.9	16.7	15.7

**Table 3:** IR data observed for *Curculionides*, *α* and *β*-chitin [39].

Functional Group	Curculionides Chitin	*α*-Chitin	*β*-Chitin
O-H stretching	3380–3450 cm^−1^	3433 cm^−1^	-
N-H stretching	3250–3300 cm^−1^	3104, 3260 cm^−1^	3269 cm^−1^
CH_3_ sym. stretch and CH_2_ asym. stretch	2850–2950 cm^−1^	2940, 2875 cm^−1^	2937, 2881 cm^−1^
C=O secondary amide stretch	1635–1660 cm^−1^	1652, 1620 cm^−1^	1640 cm^−1^
N-H bend, C-N stretch	1560–1580 cm^−1^	1552 cm^−1^	1552 cm^−1^
CH_2_ ending and CH_3_ deformation	1410–1425 cm^−1^	1420 cm^−1^	1442 cm^−1^
CH bend, CH_3_ deformation	1370–1385 cm^−1^	1375 cm^−1^	1374 cm^−1^
CH_2_ wagging	1305–1310 cm^−1^	1307 cm^−1^	1308 cm^−1^
Asymmetric brids oxygen stretching	1150–1160 cm^−1^	1154 cm^−1^	1146 cm^−1^
Asymmetric in phase ring stretching mode	1110–1115 cm^−1^	1112 cm^−1^	1110 cm^−1^
C-O-C asym. stretch in phase ring	1065–1075 cm^−1^	1067 cm^−1^	1060 cm^−1^
C-O asym. stretch in phase ring	1005–1015 cm^−1^	1008 cm^−1^	1027 cm^−1^
CH_3_ wagging	950–955 cm^−1^	951 cm^−1^	947 cm^−1^
CH ring stretching	890–900 cm^−1^	895 cm^−1^	890 cm^−1^

**Table 4:** Thermal analysis data.

Species	Thermal Degradation Temperature (°C)	Ash Content (%)	Water Content (%)
*Eupholus cuvieri*	370.5	4.0	3.8
*Eupholus magnificus*	373.7	3.7	4.0
*Lixus albicornis*	350.5	3.5	3.1
*Lixus gigas*	366.5	5.3	3.5
*Lixus sturmii*	371.5	5.0	3.5
*Holonychus saxosus*	371.8	3.9	4.2
*Pachyrhynchus reticulatus*	356.3	5.6	4.4
*Pachyrhynchus gemmatus*	368.2	9.7	1.8
*Sipalinus gigas*	369.8	4.7	4.7

**Table 5:** Elemental analysis data.

Species	C (%)	H (%)	N (%)
Theoretical value	46.8	6.5	6.8
*Eupholus cuvieri*	42.8	6.4	6.1
*Eupholus magnificus*	42.7	6.2	6.2
*Lixus albicornis*	42.6	6.3	6.2
*Lixus gigas*	41.9	6.2	6.1
*Lixus sturmii*	42.6	6.4	6.3
*Holonychus saxosus*	41.2	6.2	6.0
*Pachyrhynchus reticulatus*	42.3	6.2	6.3
*Pachyrhynchus gemmatus*	43.8	6.5	5.4
*Sipalinus gigas*	40.6	6.1	5.8

**Table 6:** XRD values observed for extracted, *α* and *β*-chitin [39].

Chitin	XRD Diffraction Pattern 2*θ*					
Curculionides chitin	9.4°	12.9°	19.6°	20.6°	22.3°	26.4°
*α*-chitin	9.46°	12.74°	19.54°	20.98°	23.08°	26.42°
*β*-chitin	8.59°	12.29°	19.62°	20.77°	22.61°	27.05°

**Table 7:** Calculated crystallinity index.

Species	Crystallinity Index (%)
*Eupholus cuvieri*	46.4
*Eupholus magnificus*	48.9
*Lixus albicornis*	59.7
*Lixus gigas*	48.3
*Lixus sturmii*	34.8
*Holonychus saxosus*	48.3
*Pachyrhynchus reticulatus*	58.7
*Pachyrhynchus gemmatus*	56.9
*Sipalinus gigas*	56.4

## Data Availability

Data is contained within the article and Supplementary File.

## Supplementary Materials

The following supporting information can be downloaded at: https://www.mdpi.com/article/10.3390/biomimetics9100608/s1, Figure S1. Examples of SEM images (scale bar = 30 µm) observed for raw surfaces of *E. cuvieri* (A and B), *E. magnificus* (C and D).; Figure S2. Examples of SEM images (scale bar = 30 µm) observed for raw surfaces of *P. gemmatus purpureus* (A and B) and *P. reticulatus* (C and D); Figure S3. Examples of SEM images (scale bar = 30 µm) observed for raw surfaces of *L. sturmii* (A and B), *L. gigas* (C and D) and *L. albicornis* (E and F); Figure S4. Examples of SEM images (scale bar = 30 µm) observed for raw surfaces of *H. saxosus* (A and B) and *S. gigas* (C and D); Figure S5. FT-IR spectrum for chitin extracted from *E. cuvieri*; Figure S6. FT-IR spectrum for chitin extracted from *E. magnificus*; Figure S7. FT-IR spectrum for chitin extracted from *L. albicornis*; Figure S8. FT-IR spectrum for chitin extracted from *L. gigas*; Figure S9. FT-IR spectrum for chitin extracted from *L. sturmii*; Figure S10. FT-IR spectrum for chitin extracted from *H. saxosus*; Figure S11. FT-IR spectrum for chitin extracted from *P. reticulatus*; Figure S12. FT-IR spectrum for chitin extracted from *P. purpureus*; Figure S13. FT-IR spectrum for chitin extracted from *S. gigas*; Figure S14. Thermal analysis (TGA in red and DTG in blue) of chitin extracted from *E. cuvieri*; Figure S15. Thermal analysis (TGA in red and DTG in blue) of chitin extracted from *E. magnificus*; Figure S16. Thermal analysis (TGA in red and DTG in blue) of chitin extracted from *L. albicornis*; Figure S17. Thermal analysis (TGA in red and DTG in blue) of chitin extracted from *L. gigas*; Figure S18. Thermal analysis (TGA in red and DTG in blue) of chitin extracted from *L. sturmii*; Figure S19. Thermal analysis (TGA in red and DTG in blue) of chitin extracted from *H. saxosus*; Figure S20. Thermal analysis (TGA in red and DTG in blue) of chitin extracted from *P. reticulatus*; Figure S21. Thermal analysis (TGA in red and DTG in blue) of chitin extracted from *P. purpureus*; Figure S22. Thermal analysis (TGA in red and DTG in blue) of chitin extracted from *S. gigas*.

## References

[1] Manosathiyadevan M., Bhuvaneshwari V., Latha R., Dhanarajan A. (2017). Impact of Insects and Pests in Loss of Crop Production: A Review. Sustainable Agriculture towards Food Security.

[2] Oman P. (1968). Prevention, Surveillance and Management of Invading Pest Insects. Bull. Entomol. Soc. Am..

[3] Khoushab F., Yamabhai M. (2010). Chitin Research Revisited. Mar. Drugs.

[4] Sikorski P., Hori R., Wada M. (2009). Revisit of α-Chitin Crystal Structure Using High Resolution X-Ray Diffraction Data. Biomacromolecules.

[5] Dutta P.K., Ravikumar M.N.V., Dutta J. (2002). Chitin and chitosan for versatile applications. J. Macromol. Sci. Part C Polym. Rev..

[6] Kim I.-Y., Seo S.-J., Moon H.-S., Yoo M.-K., Park I.-Y., Kim B.-C., Cho C.-S. (2008). Chitosan and Its Derivatives for Tissue Engineering Applications. Biotechnol. Adv..

[7] Wang H., Qian J., Ding F. (2018). Emerging Chitosan-Based Films for Food Packaging Applications. J. Agric. Food Chem..

[8] Negm N.A., Hefni H.H.H., Abd-Elaal A.A.A., Badr E.A., Abou Kana M.T.H. (2020). Advancement on Modification of Chitosan Biopolymer and Its Potential Applications. Int. J. Biol. Macromol..

[9] Ali Khan Z., Jamil S., Akhtar A., Mustehsan Bashir M., Yar M. (2020). Chitosan Based Hybrid Materials Used for Wound Healing Applications- A Short Review. Int. J. Polym. Mater. Polym. Biomater..

[10] Rufato K.B., Galdino J.P., Ody K.S., Pereira A.G., Corradini E., Martins A.F., Paulino A.T., Fajardo A.R., Aouada F.A., La Porta F.A., Popa L., Violeta Ghica M., Dinu-Pîrvu C.-E. (2019). Hydrogels Based on Chitosan and Chitosan Derivatives for Biomedical Applications. Hydrogels—Smart Materials for Biomedical Applications.

[11] Shahid M., Mohammad F. (2013). Green Chemistry Approaches to Develop Antimicrobial Textiles Based on Sustainable Biopolymers—A Review. Ind. Eng. Chem. Res..

[12] Thakur V.K., Thakur M.K. (2014). Recent Advances in Graft Copolymerization and Applications of Chitosan: A Review. ACS Sustain. Chem. Eng..

[13] Oyatogun G.M., Esan T.A., Akpan E.I., Adeosun S.O., Popoola A.P.I., Imasogie B.I., Soboyejo W.O., Afonja A.A., Ibitoye S.A., Abere V.D. (2020). Chitin, Chitosan, Marine to Market. Handbook of Chitin and Chitosan.

[14] Yadav M., Goswami P., Paritosh K., Kumar M., Pareek N., Vivekanand V. (2019). Seafood Waste: A Source for Preparation of Commercially Employable Chitin/Chitosan Materials. Bioresour. Bioprocess..

[15] Uranga J., Etxabide A., Cabezudo S., de la Caba K., Guerrero P. (2020). Valorization of Marine-Derived Biowaste to Develop Chitin/Fish Gelatin Products as Bioactive Carriers and Moisture Scavengers. Sci. Total Environ..

[16] Ifuku S., Nomura R., Morimoto M., Saimoto H. (2011). Preparation of Chitin Nanofibers from Mushrooms. Materials.

[17] Vetter J. (2007). Chitin Content of Cultivated Mushrooms Agaricus Bisporus, Pleurotus Ostreatus and Lentinula Edodes. Food Chem..

[18] Juárez-de la Rosa B.A., Quintana P., Ardisson P.-L., Yáñez-Limón J.M., Alvarado-Gil J.J. (2012). Effects of Thermal Treatments on the Structure of Two Black Coral Species Chitinous Exoskeleton. J. Mater. Sci..

[19] Soon C.Y., Tee Y.B., Tan C.H., Rosnita A.T., Khalina A. (2018). Extraction and Physicochemical Characterization of Chitin and Chitosan from Zophobas Morio Larvae in Varying Sodium Hydroxide Concentration. Int. J. Biol. Macromol..

[20] Wysokowski M., Bazhenov V.V., Tsurkan M.V., Galli R., Stelling A.L., Stöcker H., Kaiser S., Niederschlag E., Gärtner G., Behm T. (2013). Isolation and Identification of Chitin in Three-Dimensional Skeleton of Aplysina Fistularis Marine Sponge. Int. J. Biol. Macromol..

[21] Kaya M., Baran T., Mentes A., Asaroglu M., Sezen G., Tozak K.O. (2014). Extraction and Characterization of α-Chitin and Chitosan from Six Different Aquatic Invertebrates. Food Biophys..

[22] Kabalak M., Aracagök D., Torun M. (2020). Extraction, Characterization and Comparison of Chitins from Large Bodied Four Coleoptera and Orthoptera Species. Int. J. Biol. Macromol..

[23] Zainol Abidin N.A., Kormin F., Zainol Abidin N.A., Mohamed Anuar N.A.F., Abu Bakar M.F. (2020). The Potential of Insects as Alternative Sources of Chitin: An Overview on the Chemical Method of Extraction from Various Sources. IJMS.

[24] Godeau X.Y., Andrianandrasana F.J., Volkova O., Szczepanski C.R., Zenerino A., Montreuil O., Godeau R.-P., Kuzhir P., Godeau G. (2022). Investigation on Dung Beetle’s (Heliocopris Hope, 1838) Chitosan Valorisation for Hydrogel 3D Printing. Int. J. Biol. Macromol..

[25] Marmier T., Szczepanski C.R., Candet C., Zenerino A., Godeau R.-P., Godeau G. (2020). Investigation on Mecynorhina Torquata Drury, 1782 (Coleoptera, Cetoniidae, Goliathini) Cuticle: Surface Properties, Chitin and Chitosan Extraction. Int. J. Biol. Macromol..

[26] Fournier P., Szczepanski C.R., Godeau R.-P., Godeau G. (2020). Chitosan Extraction from Goliathus Orientalis Moser, 1909: Characterization and Comparison with Commercially Available Chitosan. Biomimetics.

[27] Muimba-Kankolongo A. (2018). Fruit Production. Food Crop Production by Smallholder Farmers in Southern Africa.

[28] Van Huis A. (2021). Cultural Significance of Beetles in Sub-Saharan Africa. Insects.

[29] Gressitt J.L., Gressitt J.L. (1982). Ecology and Biogeography of New Guinea Coleoptera (Beetles). Biogeography and Ecology of New Guinea.

[30] Fernández D.C., VanLaerhoven S.L., McCreary C., Labbé R.M. (2020). An Overview of the Pepper Weevil (Coleoptera: Curculionidae) as a Pest of Greenhouse Peppers. J. Integr. Pest Manag..

[31] Fathi S.A.A., Abedi A.A. (2014). Ovipositional Preference and Life History Parameters of Lixus incanescens (Coleoptera: Curculionidae) on Selected Sugar Beet Cultivars. Int. J. Pest Manag..

[32] Sawicka B., Egbuna C. (2020). Pests of Agricultural Crops and Control Measures. Natural Remedies for Pest, Disease and Weed Control.

[33] Marei N.H., El-Samie E.A., Salah T., Saad G.R., Elwahy A.H.M. (2016). Isolation and Characterization of Chitosan from Different Local Insects in Egypt. Int. J. Biol. Macromol..

[34] Shin S., Clarke D.J., Lemmon A.R., Moriarty Lemmon E., Aitken A.L., Haddad S., Farrell B.D., Marvaldi A.E., Oberprieler R.G., McKenna D.D. (2018). Phylogenomic Data Yield New and Robust Insights into the Phylogeny and Evolution of Weevils. Mol. Biol. Evol..

[35] Okiwelu S.N., Ndome C.B., Ide Y.F. (1988). Insect Pests of Leafy Vegetables in Rivers State, Nigeria: I. Feeding Habits and Infestation of the Bitterleaf Weevil Lixus Camerunus Kolbe (Coleoptera: Curculionidae). Int. J. Trop. Insect Sci..

[36] Nakamura K., Lang X. (2002). Development and Survivorship of the Japanese Giant Weevil, Sipalinus Gigas(Fabricius)(Coleoptera: Rhynchophoridae), in Cut Pine Bolts. Appl. Entomol. Zool..

[37] Percot A., Viton C., Domard A. (2003). Optimization of Chitin Extraction from Shrimp Shells. Biomacromolecules.

[38] Pouya C., Stavenga D.G., Vukusic P. (2011). Discovery of Ordered and Quasi-Ordered Photonic Crystal Structures in the Scales of the Beetle Eupholus Magnificus. Opt. Express.

[39] Tsurkan M.V., Voronkina A., Khrunyk Y., Wysokowski M., Petrenko I., Ehrlich H. (2021). Progress in Chitin Analytics. Carbohydr. Polym..

[40] Sajomsang W., Gonil P. (2010). Preparation and Characterization of α-Chitin from Cicada Sloughs. Mater. Sci. Eng. C.

