# Supplementary material for: Investigation of 9 True Weevil (Curculionidae Latreille, 1802) Species for Chitin Extraction
Source: Biomimetics (Basel). 2024 Oct 8;9(10):608. doi: 10.3390/biomimetics9100608 (PMC11505005; doi:10.3390/biomimetics9100608)
Supplement: Supplementary file 1 [file biomimetics-09-00608-s001.zip › biomimetics-3217990-supplementary.pdf]

**Investigation on Curculionidae (Latreille, 1802) for chitin extraction.**

Zhenying Mei[1], Luc Vincent[2], Caroline R. Szczepanski[3], René-Paul Godeau[1], Pavel Kuzhir[1], Guilhem Godeau[1,4]*

*[1] Université Côte d'Azur, CNRS UMR 7010 INPHYNI, Parc Valrose Nice, 06108 France*

*[2] Université Côte d'Azur, CNRS UMR 7272 ICN, Parc Valrose Nice, 06108 France*

*[3]Department of Chemical Engineering and Materials Science, Michigan State University, East Lansing, MI 48824 USA*

*[4] Université Côte d'Azur, IMREDD, 06200 Nice, France*

Tel: (+33) 4 89 15 29 04

E-mail: guilhem.godeau@univ-cotedazur.fr

## Supplementary Data

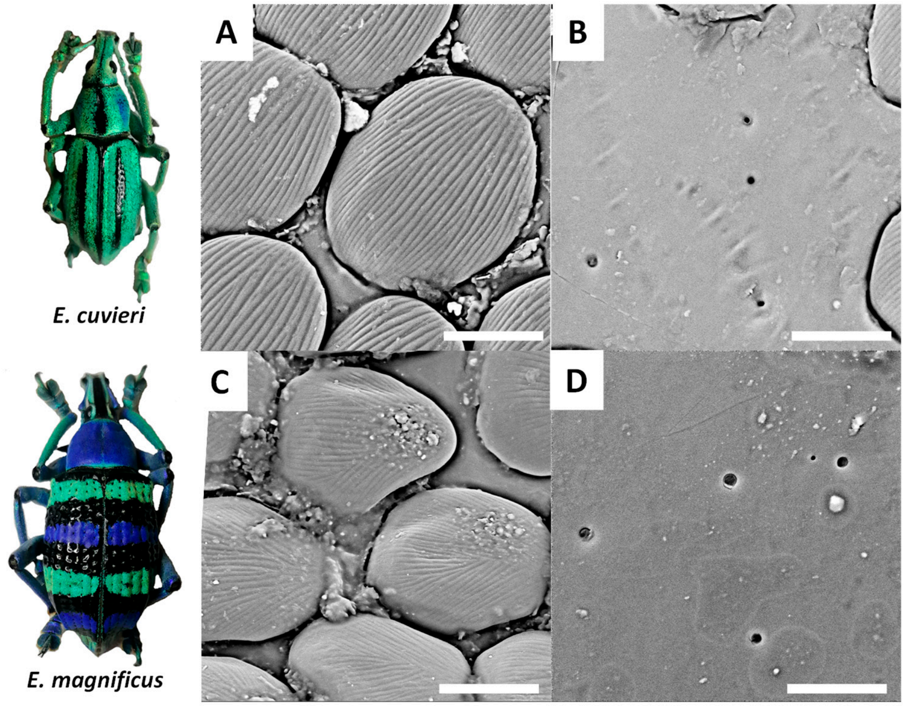


**Figure S1.** Examples of SEM images (scale bar = 30 $\mu$m) observed for raw surfaces of *E. cuvieri* (A and B), *E. magnificus* (C and D).

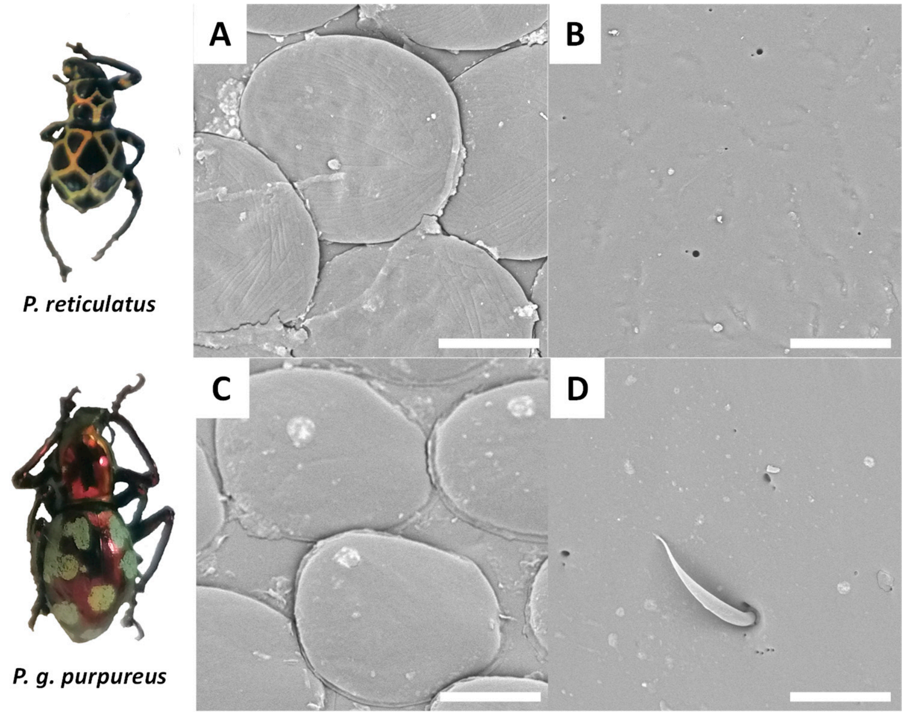


**Figure S2.** Examples of SEM images (scale bar = 30 $\mu$m) observed for raw surfaces of *P. gemmatus purpureus* (A and B) and *P. reticulatus* (C and D).

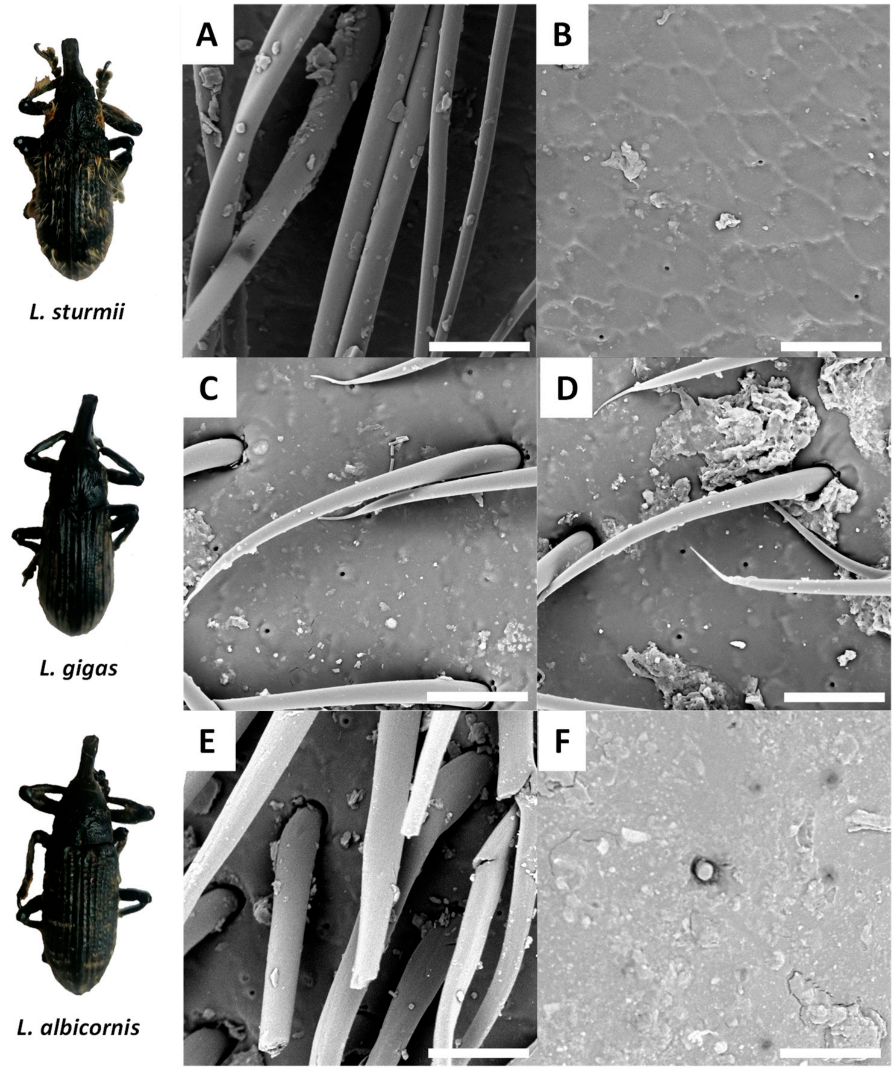


**Figure S3.** Examples of SEM images (scale bar = 30 $\mu$m) observed for raw surfaces of *L. sturmii* (A and B), *L. gigas* (C and D) and *L. albicornis* (E and F).

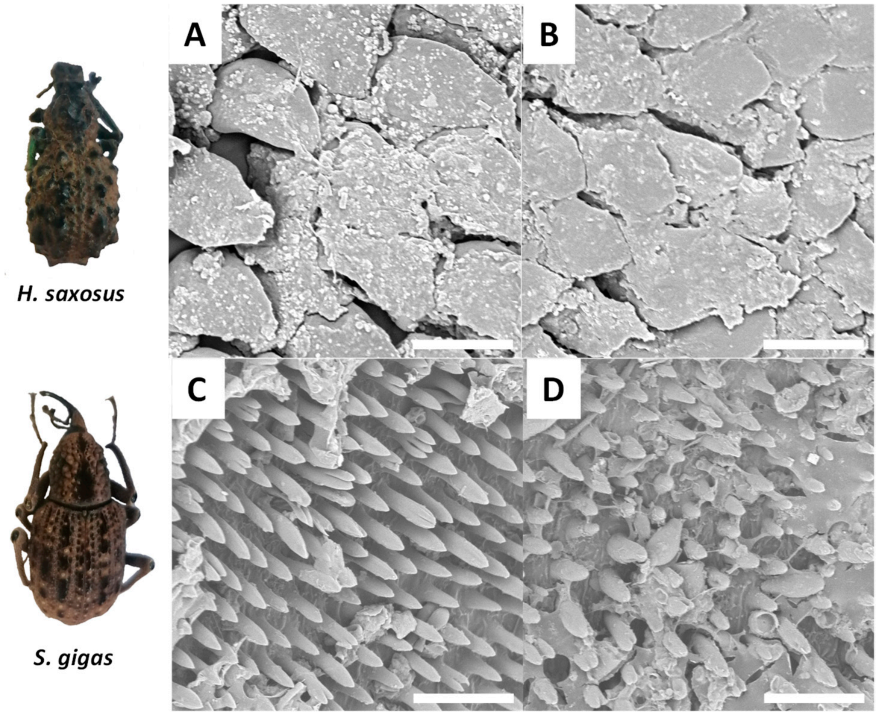


**Figure S4.** Examples of SEM images (scale bar = 30 $\mu$m) observed for raw surfaces of *H. saxosus* (A and B) and *S. gigas* (C and D).

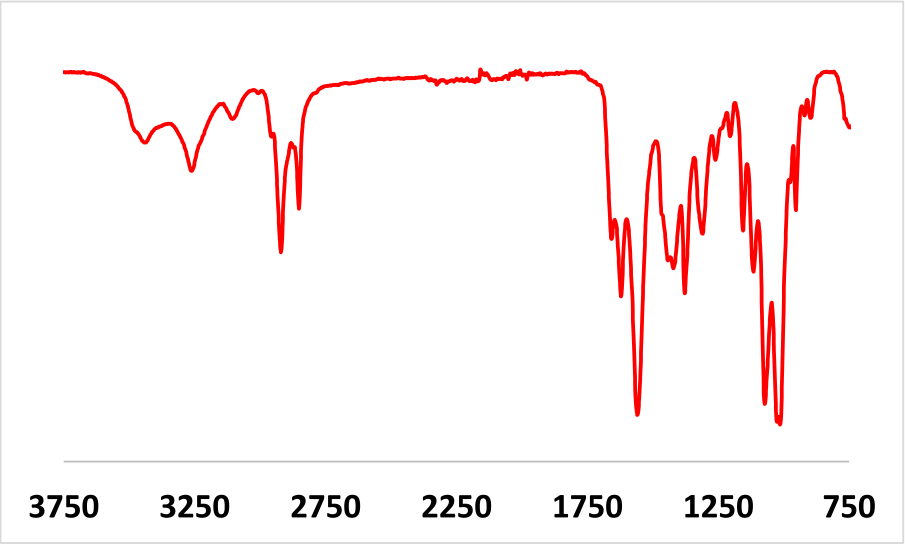


**Figure S5.** FT-IR spectrum for chitin extracted from *E. cuvieri*.

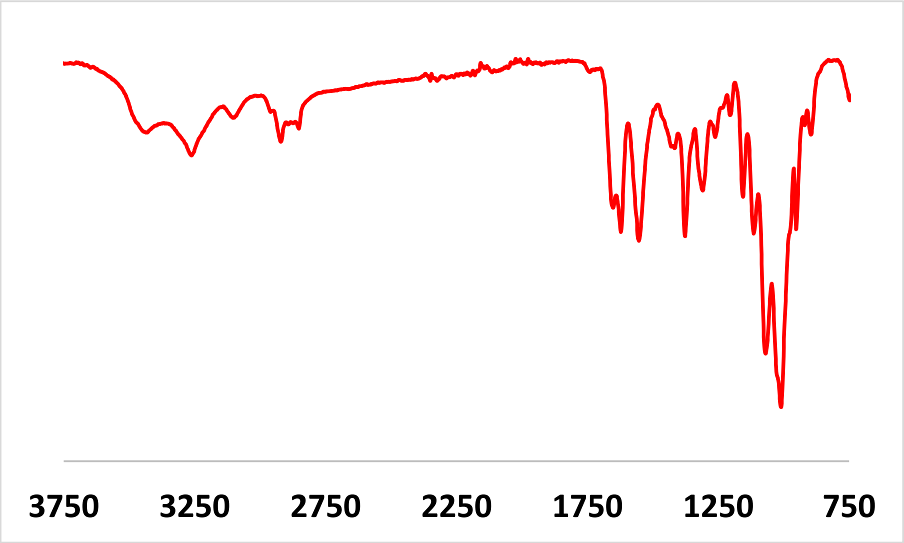


**Figure S6.** FT-IR spectrum for chitin extracted from *E. magnificus*.

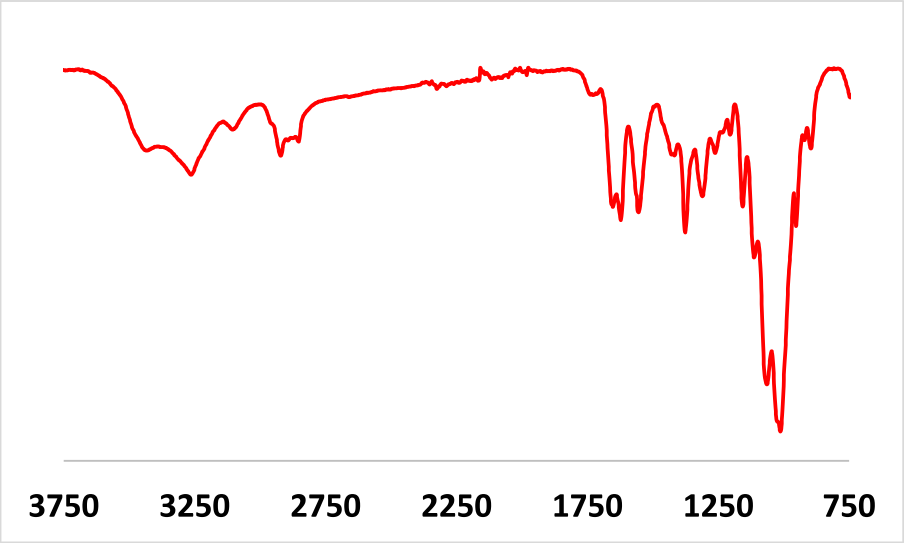


**Figure S7.** FT-IR spectrum for chitin extracted from *L. albicornis*.

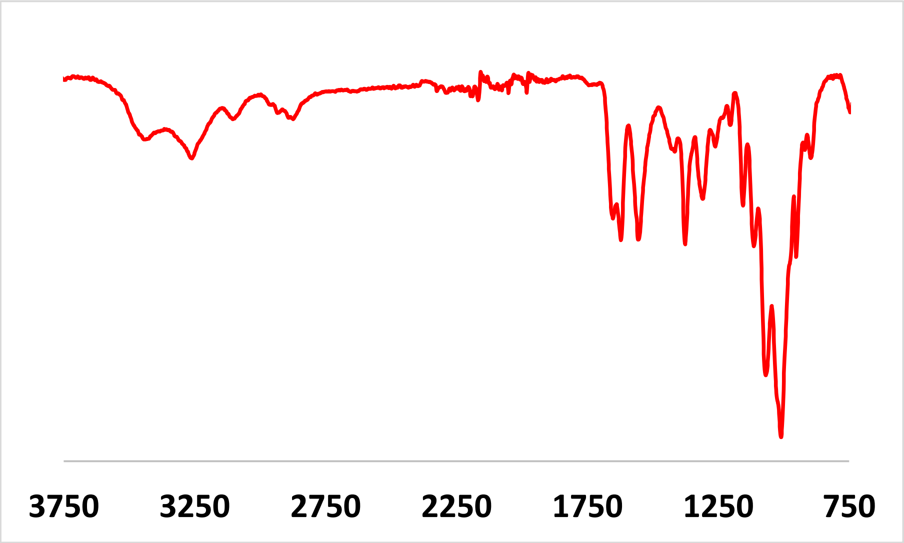


**Figure S8.** FT-IR spectrum for chitin extracted from *L. gigas*.

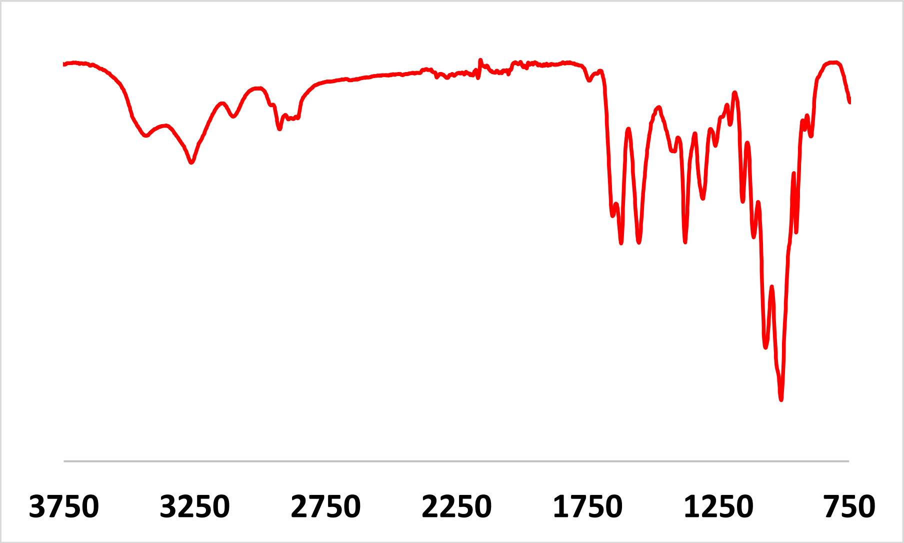


**Figure S9.** FT-IR spectrum for chitin extracted from *L. sturmii*.

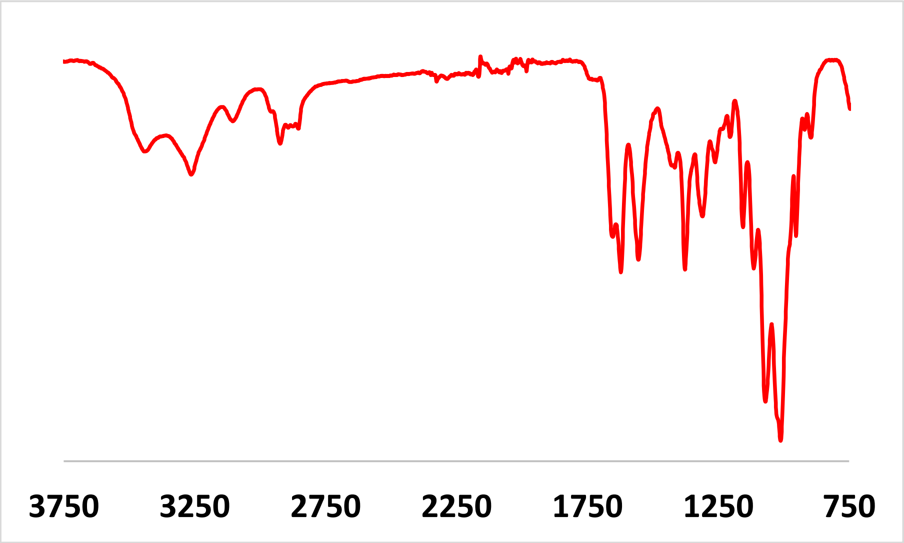


**Figure S10.** FT-IR spectrum for chitin extracted from *H. saxosus*.

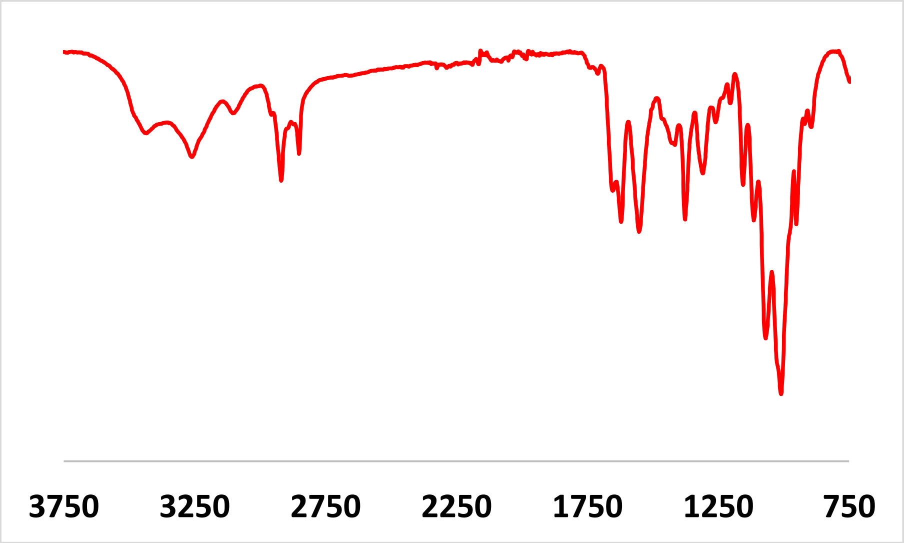


**Figure S11.** FT-IR spectrum for chitin extracted from *P. reticulatus*.

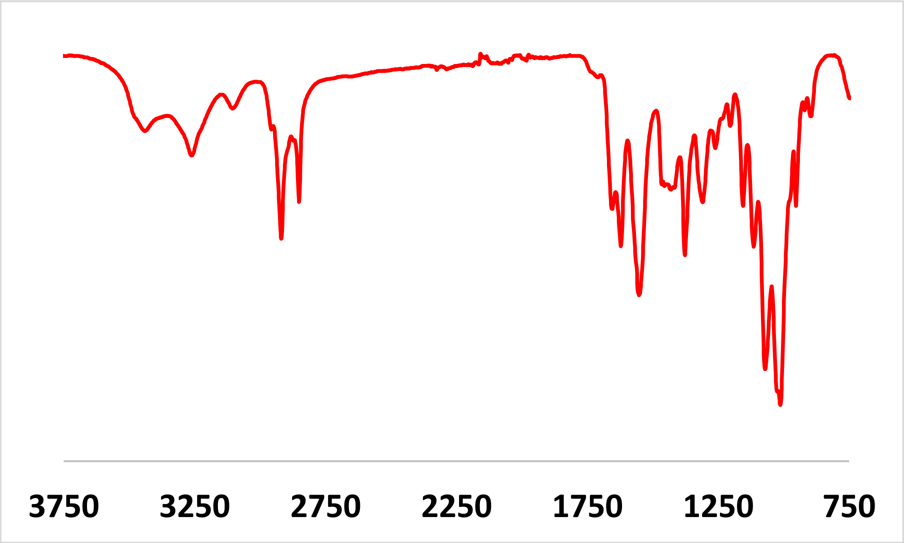


**Figure S12.** FT-IR spectrum for chitin extracted from *P. purpureus*.

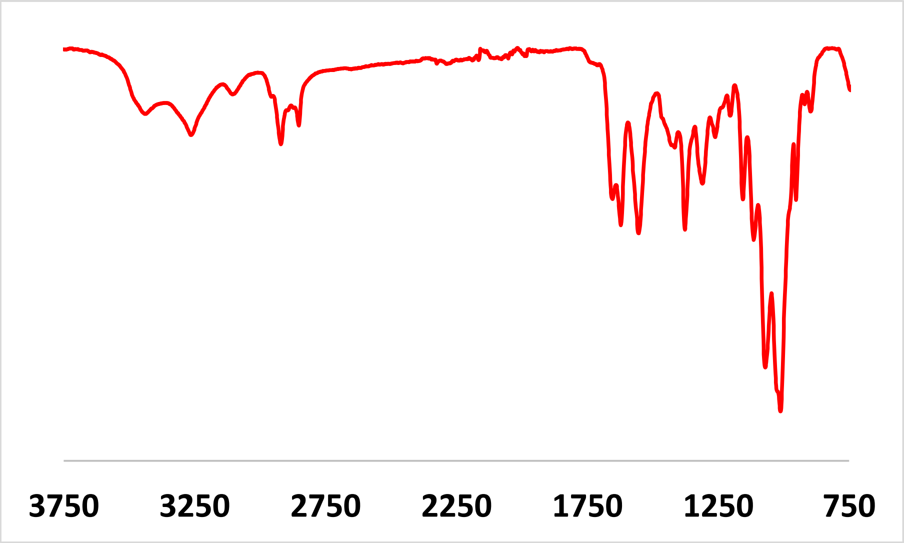

**Figure S13.** FT-IR spectrum for chitin extracted from *S. gigas*.

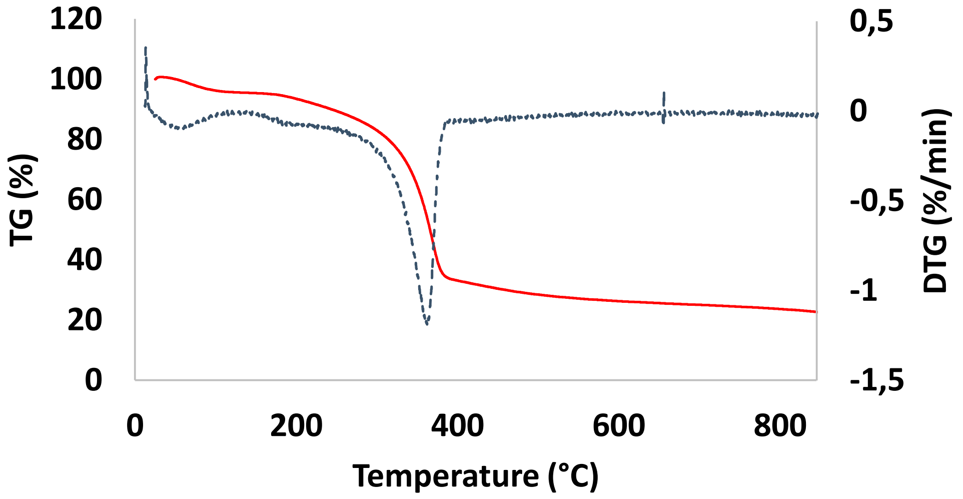


**Figure S14.** Thermal analysis (TGA in red and DTG in blue) of chitin extracted from *E. cuvieri*.

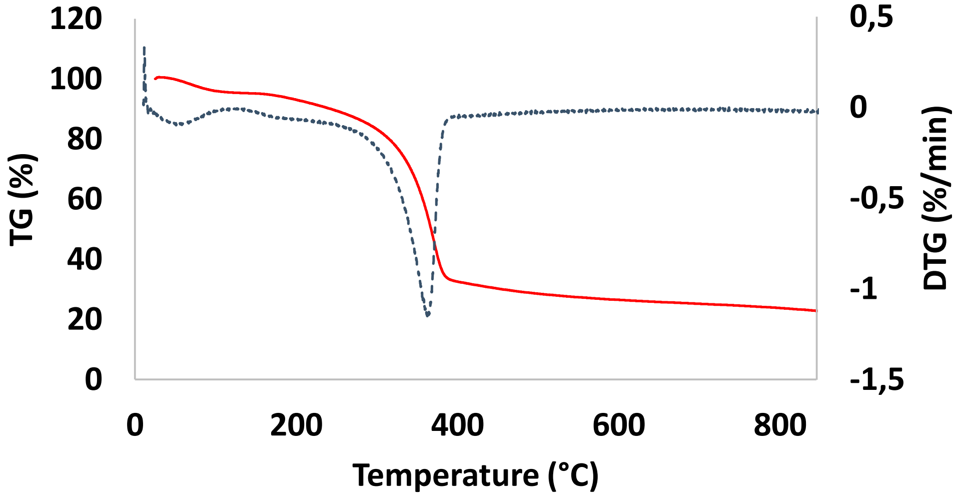


**Figure S15.** Thermal analysis (TGA in red and DTG in blue) of chitin extracted from *E. magnificus*.

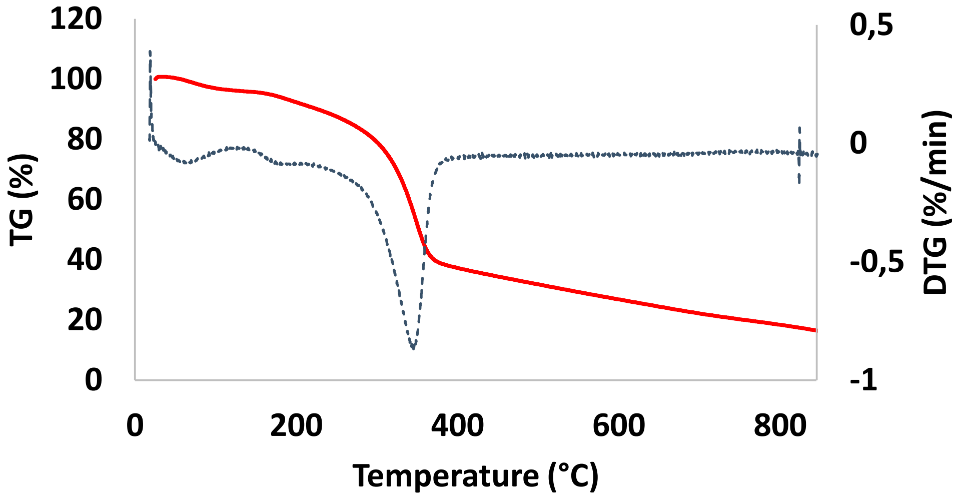


**Figure S16.** Thermal analysis (TGA in red and DTG in blue) of chitin extracted from *L. albicornis*.

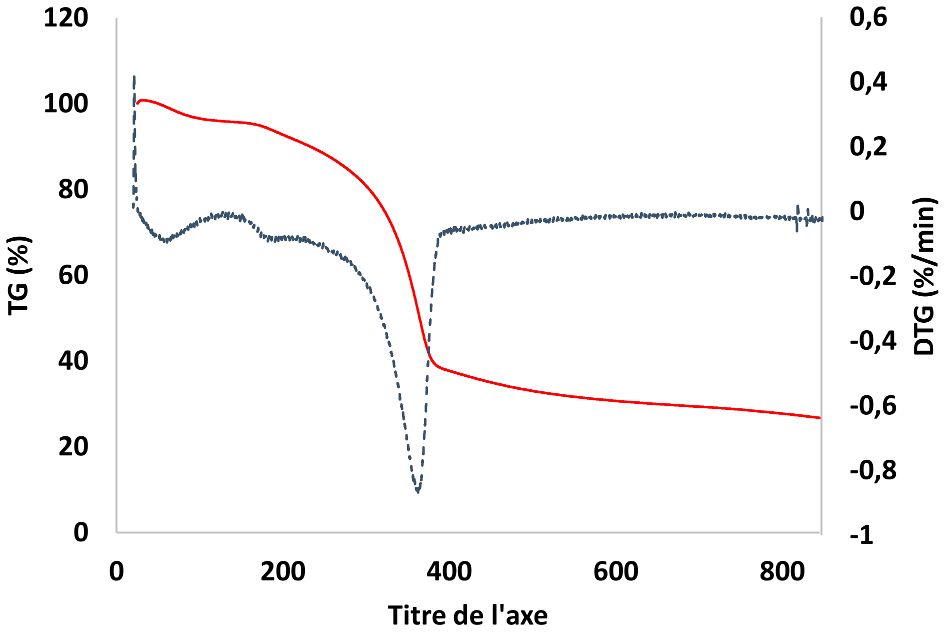


**Figure S17.** Thermal analysis (TGA in red and DTG in blue) of chitin extracted from *L. gigas*.

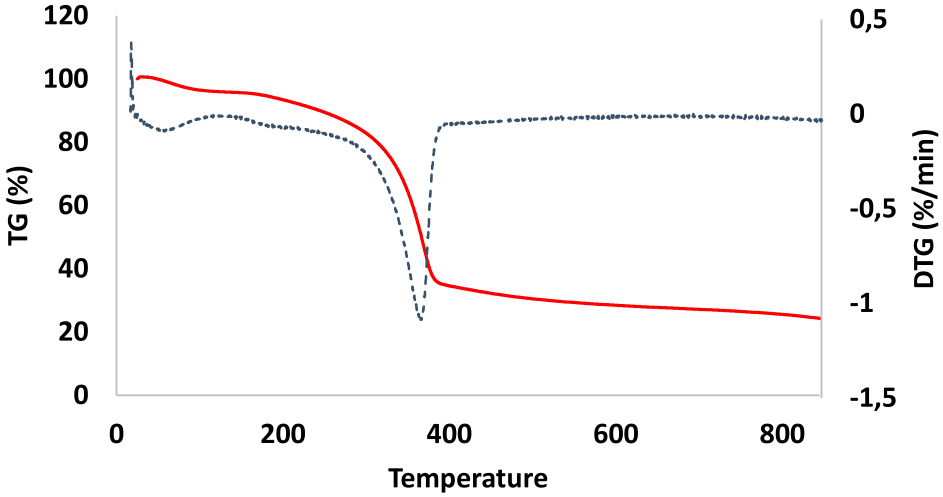


**Figure S18.** Thermal analysis (TGA in red and DTG in blue) of chitin extracted from *L. sturmii*.

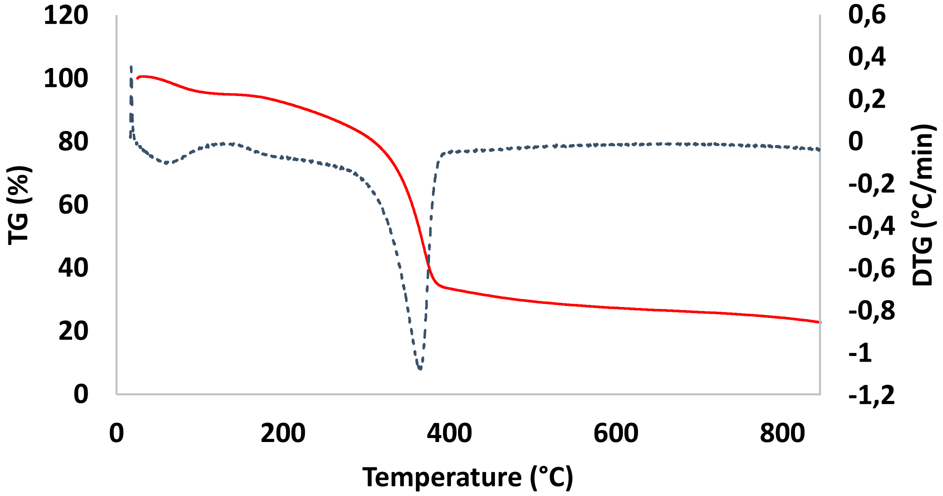


**Figure S19.** Thermal analysis (TGA in red and DTG in blue) of chitin extracted from *H. saxosus*.

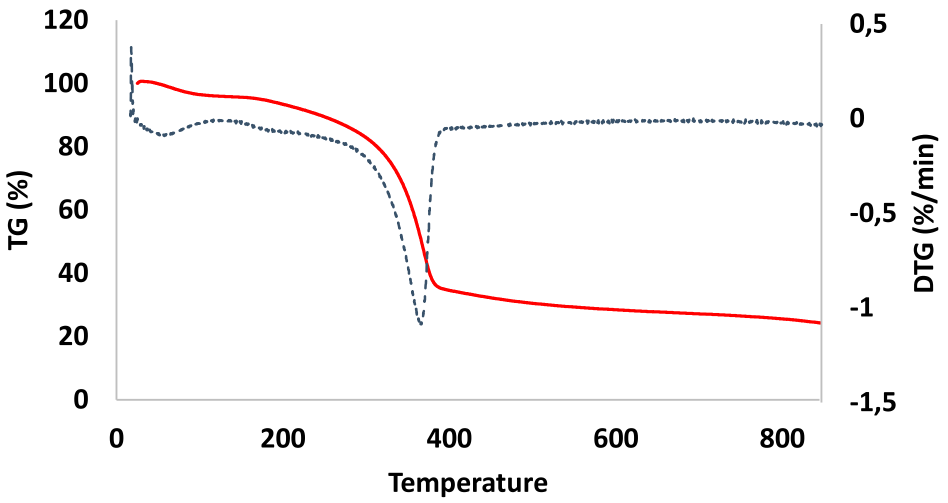


**Figure S20.** Thermal analysis (TGA in red and DTG in blue) of chitin extracted from *P. reticulatus*.

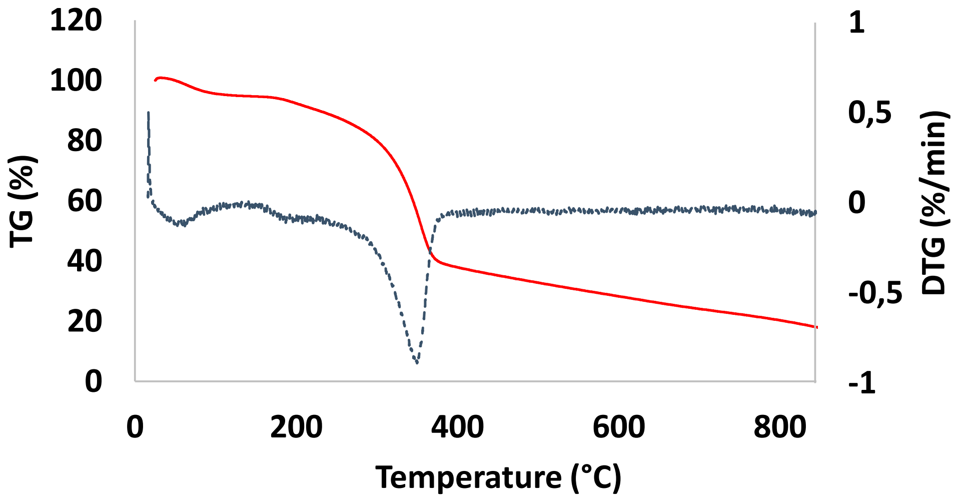


**Figure S21.** Thermal analysis (TGA in red and DTG in blue) of chitin extracted from *P. purpureus*.

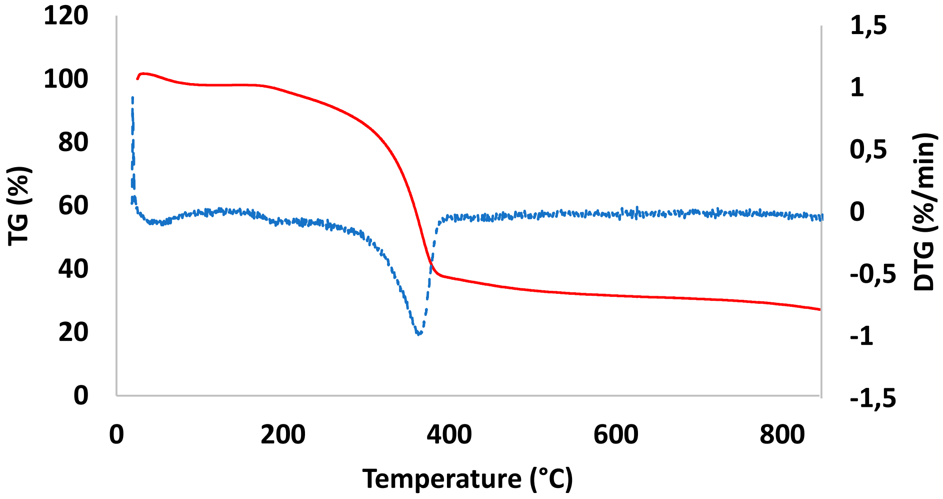


**Figure S22.** Thermal analysis (TGA in red and DTG in blue) of chitin extracted from *S. gigas*.
